# Comparative effects of alive and pasteurized *Akkermansia muciniphila* on normal diet-fed mice

**DOI:** 10.1038/s41598-021-95738-5

**Published:** 2021-09-09

**Authors:** Fatemeh Ashrafian, Shahrbanoo Keshavarz Azizi Raftar, Arefeh Shahryari, Ava Behrouzi, Rezvan Yaghoubfar, Arezou Lari, Hamid Reza Moradi, Shohreh Khatami, Mir Davood Omrani, Farzam Vaziri, Andrea Masotti, Seyed Davar Siadat

**Affiliations:** 1 Microbiology Research Center (MRC), Pasteur Institute of Iran, Tehran, Iran; 2 Clinical Research Department, Pasteur Institute of Iran, Tehran, Iran; 3 Department of Microbiology, Faculty of Advanced Science and Technology, Tehran Medical Science, Islamic Azad University, Tehran, Iran; 4 Systems Biomedicine Unit, Pasteur Institute of Iran, Tehran, Iran; 5 Department of Basic Sciences, School of Veterinary Medicine, Shiraz University, Shiraz, Iran; 6 Biochemistry Department, Pasteur Institute of Iran, Tehran, Iran; 7 Department of Medical Genetics, Faculty of Medicine, Shahid Beheshti University of Medical Sciences, Tehran, Iran; 8 Department of Mycobacteriology and Pulmonary Research, Pasteur Institute of Iran, Tehran, Iran; 9 Research Laboratories, Children’s Hospital Bambino Gesù-IRCCS, Rome, Italy

**Keywords:** Microbiology, Diseases

## Abstract

Recently, *Akkermansia muciniphila* an anaerobic member of the gut microbiota, has been proposed as a next-generation probiotic. The aim of this study was evaluation of the effect of alive and pasteurized *A. muciniphila* on health status, intestinal integrity, immune response, lipid metabolism, and gut microbial composition in normal-diet fed mice as well as direct effects of the bacterium on Caco-2 cell line. A total of 30 mice were distributed into three different groups, control, alive, and pasteurized *A. muciniphila*-treated group. After acclimation, control and treatment groups were administrated with PBS and 10^9^ CFU/200µL of bacterial suspension for 5 weeks, respectively. Besides, Caco-2 separately exposed to alive, pasteurized *A. muciniphila* and PBS for 24 h. The results showed that administration of *A. muciniphila* leads to reduction in body, liver, and white adipose weight. Histology data revealed both treatments had no adverse effects in colon, liver, and adipose tissues as well as induced better gut structure. Moreover, biochemical parameters and inflammatory biomarkers in plasma demonstrated that pasteurized *A. muciniphila* had more pronounce effect. Furthermore, alive *A. muciniphia* had better effects on the modulation of gene expression related to fatty acid synthesis, energy homeostasis, and immune response in the liver; meanwhile, these effects in the adipose was more in the pasteurized *A. muciniphila* administration. More importantly, the improvement of gut health by enhancing strengthen intestinal integrity and maintaining immune homeostasis was seen in both treatments; notably, pasteurized *A. muciniphila* had more effective. Similarly, treatment with the pasteurized form more effectively upregulated tight junction and regulated immune response-related genes in Caco-2 cell line. Both treatments triggered the improvement of microbiota communities, particularly the alive form. Therefore, both forms of *A. muciniphila* could modulate lipid and immune homeostasis, improved some gut microbiota, and promoted the overall health, while all these effects were dominantly observed in pasteurized form. In conclusion, pasteurized *A. muciniphila* can be considered as new medical supplement to maintain health state and prevent diseases in normal mice through different mechanisms.

## Introduction

Trillions of microorganism reside in the gastrointestinal (GI) tract, known as “intestinal microbiota”, that are involved in the regulation of food intake, motility of the gut, immune and metabolic pathways^1^. In addition, numerous studies show that intestinal microbiota also plays an important role in maintaining intestinal homeostasis by improvement of the gut barrier function and the inflammatory state^2,3^. As a bilateral relationship exists between the gut microbiota and intestinal immune system, the immune system playing a crucial role in maintaining the microbiota's homeostasis, as well as the intestinal microbiota affect immune system by influencing on T-reg cells. This two-way communication is accomplished through various mechanisms, including TLRs^4^.

Disruption of intestinal microbiota composition known as “dysbiosis” has been reported in many diseases and its return to normal pattern has been associated with the amelioration of diseases^5, 6^. One of the factors contribute to restoration of healthy condition is prebiotics, probiotics, and para-probiotics consumption^7–9^.

One of the next-generation probiotics considered to be a healthy biomarker bacterium in the human and animal intestines is *Akkermansia muciniphila* (*A. muciniphila*)^10,11^*.* Many researchers have confirmed a reduction in the bacterium frequency associated with several diseases^12–14^. On the other hand, a high abundance of *A. muciniphila* is linked to decreased blood lipids and improved metabolic features that ultimately lead to a healthy state in obese adults^15^. In addition, *A. muciniphila* can affect metabolic modulation, immune response regulation, serotonergic system, and gut health maintenance^16–21^. Increased *A. muciniphila* may be associated with increased intestinal integrity and normal mucus production in the intestines of healthy individuals^22^. Several lines of evidence have indicated that *A. muciniphila*, as a closer epithelial bacterium, have an impact on health promotion^7,23^.

In comparison with viable probiotics, para-probiotics are non-viable and have more effective properties^9,17^. Researchers reported that probiotics and para-probiotics have an important role in restoring health and ameliorating diseases^12,17,18,24,25^. But limited research has been performed on whether they can also be effective in maintaining health in normal subjects or animals and further studies are needed for detecting precise molecular mechanism in this area.

In the present study, the effects of alive and pasteurized *A. muciniphila* on the integrity of gut barrier, inflammation, and energy homeostasis were assessed in vitro and in vivo. Our research provided an insight into the beneficial effects of *A. muciniphila* on host health and the safety of its use in normal diet-fed mice.

## Material and method

### Bacterial culture and pasteurization

*A. muciniphila* Muc^T^ (ATCC BAA-835) was cultured in a synthetic medium under the anaerobic conditions as previously described^17^. After growth, the bacterium was inoculated into the broth with mild shaking under the above-mentioned conditions. After OD_600_ was reached 1, bacterial pellets were collected by centrifugation and then washed twice with an anaerobic PBS. After resuspended in PBS, the suspension used for the treatments was prepared freshly. For the preparation of pasteurized form, the bacteria suspension was heated at 70 °C for 30 min^17^.

### Cell culture conditions, treatment, and quantitative real-time PCR

Human colorectal carcinoma cell line Caco-2 (ATCC® HTB-37) was cultured in DMEM (Gibco, UK), supplemented with 10% heat-inactivated FBS (Gibco) and 1% penicillin–streptomycin (Gibco) at 37 °C in 5% CO_2_. After 21 days of culture, Caco-2 monolayer was exposed to a live and pasteurized *A. muciniphila* at Multiplicity of infection (MOI) ratio of 100 (bacteria per cell) as well as an equal volume of PBS was used as a control for bacterial treatment. RNeasy Plus Mini kit (Qiagen, USA, Cat No. /ID: 74,134), PrimeScript RT Reagent Kit (Takara, Japan, Cat. # RR037A), and 2X SYBR Premix Ex Taq II (Tli RNase H, Plus Takara, Japan, Cat. #RR820L) were used for RNA extraction, cDNA synthesis, and real-time PCR, respectively. A sequence of primers used in this study is shown in Supplementary Table 1.

### Experimental design and samples collection

All animal studies were carried out in compliance with the ARRIVE guidelines. All Animal procedures were approved by the Animal Experiment Committee of the Pasteur Institute of Iran (IR.PII.REC.1395.010) and confirming that all experiments were performed in this study in accordance with relevant guidelines and regulations. Eight-week-old mice purchased from Pasteur Institute of Iran (n = 30) and maintained on an equal condition including 12 h light/dark, 22 (± 2) °C and 40–60% humidity as well as received food and autoclaved water ad libitum. After acclimation with standard Normal Diet (ND) (A03, safe diet, France), mice were randomly divided into three groups as follows: The mice were treated for five weeks includes (1) ND + 200 µl PBS (C), (2) ND + 10^9^ CFU /200 µl alive *A. muciniphila* (Am), and (3) ND + 10^9^ CFU /200 µl pasteurized *A. muciniphila* (PAm).

The mice were individually housed in per cages and also body weight and average food were measured once a week. At the end of treatment, blood sample was collected by cardiac puncture at 12 h fasting condition and stored at  − 80 °C for biochemical plasma analysis. In addition, stool samples were collected from individually housed mice and transferred to − 80 °C. All mice were sacrificed by cervical dislocation, and liver, epididymal adipose, and colon samples snap-frozen with liquid nitrogen stored in − 80 °C for real-time PCR. Moreover, the tissue specimens are saved for histological staining.

### Plasma biochemical and cytokines analysis

Fasting blood glucose (Glu), total cholesterol (TC), triglycerides (TG), low-density lipoprotein (LDL), high-density lipoprotein (HDL), triglyceride (TG), alanine aminotransferase (ALT), and aspartate aminotransferase (AST) concentration were measured in plasma using a commercial kit (Bioclin-Quibasa, Belo Horizonte, MG, Brazil).

The concentration of IL-6, TNF-α, and IL-10 in the plasma of mice were determined by using ZellBio GmbH ELISA kit (Germany) according to the manufacturer’s instructions.

### Histological evaluation

Four samples from each group were used for pathology examination. The colon, liver and adipose tissues were immersed in 10% buffered formalin. Then, tissues were dehydrated in ascending graded series of ethanol. They cleared in xylene and impregnated and embedded in paraffin. Paraffin blocks were cut using microtome at 5–7 µm thickness and mounted on glass slides. Histological sections were stained with hematoxylin and eosin (H&E). Then, histological slides were evaluated using a light microscope (Olympus SX-21) equipped with a digital Dino-Lite lens and Dino-capture 2 software (AnMo Electronics Corp., New Taipei City, Taiwan)^26^. Stained sections were evaluated by an expert pathologist, blind to study groups.

### Tissue RNA isolation, cDNA synthesis, and real-time PCR

The liver, colon, and adipose were homogenized and RNA was extracted by Trizol reagent (Bio Basic, Canada). Genomic DNA removed from RNA by DNase I (Qiagen) then cDNA synthesis was performed using PrimeScript RT Reagent Kit (Takara). Real-time PCR was performed using SYBR Premix Ex Taq II (Takara). A sequence of primers used in this study is shown in Supplementary Table 1.

### Bacterial DNA extraction and real-time quantitative PCR

Each stool sample was weighted at 180 mg and homogenized, then bacterial genomic DNA was extracted using a QIAamp Fast DNA Stool Mini Kit (Qiagen, USA, Cat No. /ID:51604) according to the manufacturer's instructions. Finally, gDNA of samples were stored at − 20 °C. Real-time PCR was carried out by RealQ Plus Master Mix Green (Amplicon, Denmark). The ΔC_T_ method was used to measure each primer efficiency. Conversion of C_T_ value to a percentage of bacterial communities was performed by using percentage formula as previously described^27^. A sequence of primers used in this experiment is shown in Supplementary Table 2.

### Statistical analysis

Differences between groups were calculated using one-way analysis of variance (ANOVA) followed by Tukey’s post hoc test for multiple comparisons between more than two groups. The relative gene expression was analyzed by ΔΔCT method and internal controls were used in Caco-2, colon, liver, and adipose tissues are included *gapdh*, *rpl-19,* and *hprt-1,* respectively. For analysis of the relative difference of gene expression among treatments and control groups in both cell line and mice as well as generation of figures, GraphPad Prism 8.0 (GraphPad Software Inc, CA, USA) was used. All data are expressed as mean ± SD. In figures, data with * and ** are significantly different at *p* < 0.05 and *p* < 0.01, respectively. The non-parametric Kruskal–Wallis tests used for pairwise comparison of mean relative percentages of 16S rRNA genes between experimental groups, and *p* values less than 0.05 were taken as statically significant.

## Results

### *A. muciniphila* improved the weight of body and metabolic organs

Because the correlation between increased BMI and various diseases, prevention of body weight gain can play an effective role in promoting health status and inhibiting diseases, we evaluated the effects of alive and pasteurized *A. muciniphila* on body and metabolic organs (i.e. liver and eWAT) weight in study groups. Our finding revealed that the administration of pasteurized *A. muciniphila* caused a higher reduction in body weight gain in comparison with the alive form (*p* value 0.002 and 0.03, respectively), however, a significant difference was not observed between them. Mice were treated with both forms of *A. muciniphila* showed a slight decrease in food intake, which was not significant compared to control group. The adipose weight significantly reduced in both Am and PAm groups (*p* value 0.01 and 0.04, respectively), compared to C group. On the other hand, the liver weight of alive *A. muciniphila*-treated mice was significantly decreased (*p* value 0.0002), while it was not significant in PAm group) Table 1). Taken together, mice gavaged by alive and pasteurized *A. muciniphila* exhibited favorable effects on ameliorating body and metabolic organs weight, which indicates the beneficial role of this next generation probiotic candidate in preserving healthy status.

**Table 1:** The effect of a live and pasteurized *A. muciniphila* administration on body, adipose and liver weight, food intake, and blood parameters in ND-fed mice after 5 weeks (n = 10 for each group).

Variable	Study groups			*P* value		
	C	Am	PAm	Am vs. C	PAm vs. C	Am vs. PAm
Body weight gain (g)	2.314 ± 0.414	1.800 ± 0.321	1.561 ± 0.293	**0.031**	**0.002**	0.42
Daily food intake/mouse (g)	3.65 ± 0.512	3.614 ± 0.241	3.714 ± 0.203	0.97	0.94	0.85
Adipose weight (g)	0.240 ± 0.053	0.170 ± 0.037	0.185 ± 0.023	**0.01**	**0.04**	0.74
Liver weight (g)	1.108 ± 0.037	0.908 ± 0.084	1.033 ± 0.086	**0.0002**	0.165	**0.014**
Glu (mg/dl)	78 ± 6.75	58 ± 5.56	47 ± 6.37	** < 0.0001**	** < 0.0001**	**0.018**
TG (mg/dl)	80.13 ± 6.61	67.86 ± 6.20	66.57 ± 6.70	**0.006**	**0.002**	0.92
TC (mg/dl)	71.79 ± 9.18	67.57 ± 5.12	45.14 ± 3.30	0.51	** < 0.0001**	** < 0.0001**
LDL (mg/dl)	13.9 ± 4.56	17.04 ± 3.46	7.32 ± 1.70	0.24	**0.039**	**0.0003**
HDL (mg/dl)	51.76 ± 9.36	105.7 ± 11.18	53.29 ± 9.55	** < 0.0001**	0.95	** < 0.0001**
ALT (U/dl)	65.00 ± 6.83	65.00 ± 9.73	24.86 ± 5.17	> 0.999	** < 0.0001**	** < 0.0001**
AST (U/dl)	87.97 ± 5.29	48.14 ± 6.36	31.86 ± 4.67	** < 0.0001**	** < 0.0001**	** < 0.0001**
TNF-α (ng/L)	162.3 ± 6.22	145.20 ± 5.76	128.40 ± 6.87	**0.009**	** < 0.0001**	**0.01**
IL-6 (pg/ml)	177.60 ± 10.95	117.3 ± 12.43	89.95 ± 9.15	** < 0.0001**	** < 0.0001**	**0.015**
IL-10 (pg/ml)	349.80 ± 28.34	567.70 ± 54.69	426.50 ± 20.89	** < 0.0001**	**0.043**	**0.001**

C; control, Am; *A. muciniphila*, PAm; pasteurized *A. muciniphila,* Glu; Glucose, TG; Triglyceride, TC; Total cholesterol, LDL; low-density lipoprotein, HDL; high-density lipoprotein, ALT; alanine aminotransferase, AST; aspartate aminotransferase, TNF-α; Tumour necrosis factor-α, IL-6; Interleukin-6, and IL-10; Interleukin-10. Bold *P* value are indicated statically significant.

### *A. muciniphila* ameliorates the biochemical and inflammatory parameters

Due to the association of dysregulated lipids profile and metabolic parameters with various diseases and the importance of a balanced abovementioned markers in disease-resistant, we assessed the changing level of lipid profiles, glucose, and hepatic enzymes in the plasma of mice treated with *A. muciniphila*. A tendency to decrease biochemical parameters level after both treatments were observed, which accompanied by regulating lipid profiles i.e. reducing the level of TG, TC, and LDL and raising HDL as well as decreasing Glu, ALT, and AST. The significantly lower concentrations of TC, LDL, Glu, ALT, and AST were seen in PAm group (*p* value < 0.0001, 0.03, < 0.0001, < 0.0001, and < 0.0001, respectively). However, significant alterations in TC, LDL, and ALT levels not observed in Am group compared to C group. In addition, both treatments reduced TG concentration as compared to C group, but there was no difference between Am and PAm groups. In comparison with C group, increased HDL level was observed in Am group, while no change was seen following pasteurized *A. muciniphila*.

Both forms of *A. muciniphila* decreased TNF-a and IL-6 and also increased regulatory cytokines IL-10 level. As shown in Table 1, more reduction in concentrations of TNF-α and IL-6 was observed in PAm group (*p *value < 0.0001 and < 0.0001, respectively), while in case of IL-10, alive *A. muciniphila* had a better effect (*p *value < 0.0001). Overall, these results indicated that pasteurized *A. muciniphila* intervention promoted health and prevented the onset of metabolic disorders by lowering effects on lipid profiles, glucose, liver injury-related enzyme, and inflammatory biomarkers in the plasma of study groups.

### *A. muciniphila* caused intestinal and immune homeostasis by improving intestinal barrier function and alleviating inflammation

**Figure 1:**
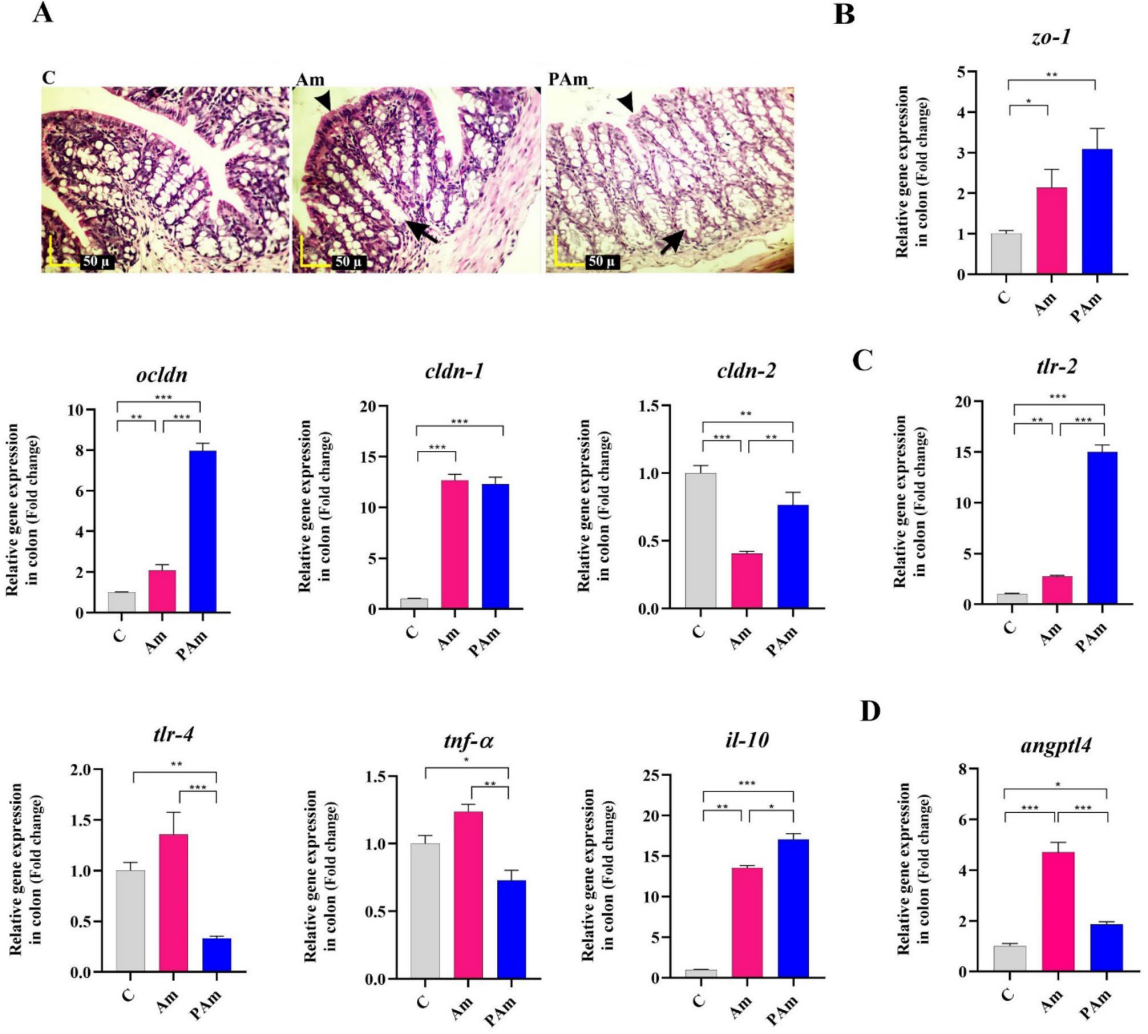
The assessment of a live and pasteurized *A. muciniphila* effects on mRNA expression of genes in the colon of ND-fed mice. Mice were gavaged with alive and pasteurized *A. muciniphila* (10^9^ CFU) for 5 weeks. (**A**) Histopathology of colon (Black arrows: crypt depth, and black arrowheads: mucous thickness) (4 samples per group). Scale bar is 50 µm. Expression of (**B**) tight junction proteins (*zo-1*, *ocldn*, *cldn-1*, and *cldn-2*), (**C**) inflammation-related genes (*tlr-2*, *tlr-4*, *tnf-α,* and *il-10*), and (**D**) *angptl4*. *, **, *** '*P* < 0.05, *P* < 0. 01, and *P* < 0. 001 were considered statistically significant, respectively. C: normal diet + PBS, Am: normal diet + *A. muciniphila* (10^9^ CFU), PAm: normal diet + pasteurized *A. muciniphila* (10^9^ CFU).

Since gut is connected to many organs in the whole body and the disruption of intestinal integrity causes inflammation and diseases, so maintaining intestinal homeostasis plays an important role in preventing the onset of various diseases. Therefore, we assessed the effects of alive and pasteurized *A. muciniphila* on morphology and intestinal barrier-related genes in the colon of ND-fed mice. The histopathological results indicated that the crypt depth and thickness of the mucous layer of the colon showed an increase in both treatment groups, compared to that in the control group. On the other hand, no inflammatory reactions were present in both study groups (Fig. 1A). Moreover, both treatments improved gut barrier function in mice through increasing tight junction proteins i.e. *zo-1*, *ocldn*, and *cldn-1* and also decreasing *cldn-2* mRNA level. The expression of *zo-1* and *cldn-1* genes significantly increased after both administrations. The significantly higher induction of down-regulating *cldn-2* was seen in Am group (*p* value < 0.0001). In addition, pasteurized *A. muciniphila* induced the expression of *ocldn* significantly more than Am group (*p *value < 0.0001 and 0.006, respectively) (Fig. 1B).

To determine whether para-probiotic and probiotic of *A. muciniphila* can affect intestinal immune homeostasis in non-inflammatory conditions, we were used mice undergoing standard diet. A significant alleviation in gene expression of *tlr-4* and significant upregultion in *tlr-2* were observed in the colon of pasteurized *A. muciniphila*-gavaged mice, compared to control group (*p* value 0.02 and < 0.0001, respectively). However, alive *A. muciniphila* consumption significantly increased the *tlr-2* mRNA level (*p* value 0.003), it was lower than that of pasteurized form. A slight increase of *tlr-4* mRNA level was observed in Am group in comparison with control group. The gavage with pasteurized *A. muciniphila* displayed a lower mRNA level of *tnf-α*, while alive form didn’t show significant change. Administrating mice with both treatments increased *il-10* expression than ND-fed mice, while PAm group was more noticeable (*p *value < 0.0001) (Fig. 1C).

To investigate whether the next-generation probiotic *A. muciniphila* affects the digestion of intestinal lipids, we evaluated *angptl4* mRNA level in the normal colon. A trend towards an increase in colonic *angptl4* was observed in both treatment groups than control group. Interestingly, alive *A. muciniphila* significantly induced a higher upregulation of *angptl4* in the healthy colon, compared to pasteurized form (*p *value < 0.0001 and 0.01, respectively) (Fig. 1D). Overall, these results showed that pasteurized *A. muciniphila* is involved in the host immunological and intestinal homeostasis at the gut by improving the function of gut barrier and regulating immune response.

### Treatment with *A. muciniphila* affect tight junction proteins and inflammatory markers in Caco-2

**Figure 2:**
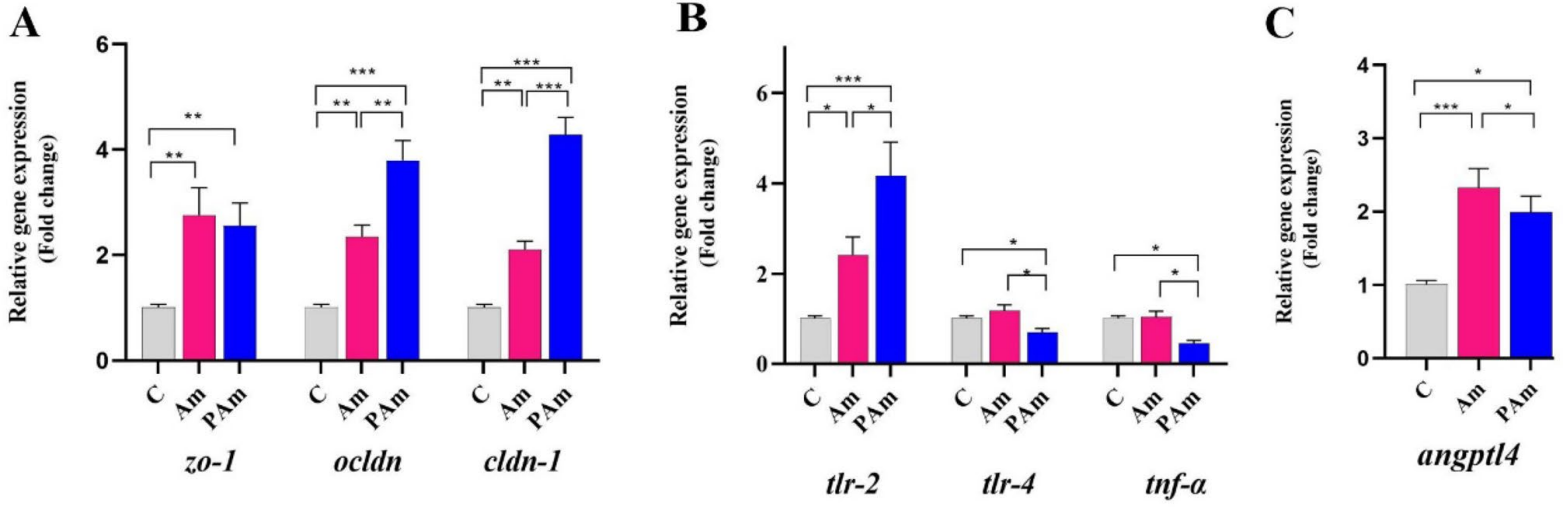
The live and pasteurized *A. muciniphila* affects study's genes in Caco-2 cell line. Caco-2 monolayer were inoculated with *A. muciniphila* (MOI_100_) and pasteurized *A. muciniphila* (MOI_100_) for 24 h. The expression of genes: (**A**) tight junction proteins (*zo-1*, *ocldn*, and *cldn-1*), (**B**) inflammation-related genes (*tlr-2*, *tlr-4*, and *tnf-α*), and (**C**) *angptl4*. *, **, *** '*P* < 0.05, *P* < 0. 01, and *P* < 0. 001 were considered statistically significant, respectively.

Caco-2 cell line was used to investigate direct effects of live and pasteurized *A. muciniphila* on the intestinal barrier function and inflammation in intestinal epithelial cells. Gene expression analysis showed tight junction proteins were upregulated, similar to mice's colon. Notably, *ocldn* and *cldn-1* to be expressed at higher amounts in PAm than Am group. Compared to vehicle-treated Caco-2 cells, both treatments showed a significant increase in mRNA level of *zo-1* (Fig. 2A). Similar to the in-vivo study, pasteurized *A. muciniphila* improved immune response, which was accompanied by down-regulating gene expression of *tlr-4* and *tnf-α* as well as up-regulating *tlr-2* genes (*p *value 0.02, 0.01, and 0.0005, respectively), while no change in *tlr-4* and *tnf-α* expression was observed in Am group (Fig. 2B). In addition, the regulation of lipid metabolism by up-regulating *angptl4* was observed after both treatments, whereas Am group was more noticeable (*p *value 0.0005) (Fig. 2C). Overall, these results suggested that alive and pasteurized *A. muciniphila* had a direct effect on the gene expression involved in integrity, inflammation, and lipid metabolism.

### Administration of *A. muciniphila* improved liver health by reducing lipid metabolism and inflammation

**Figure 3:**
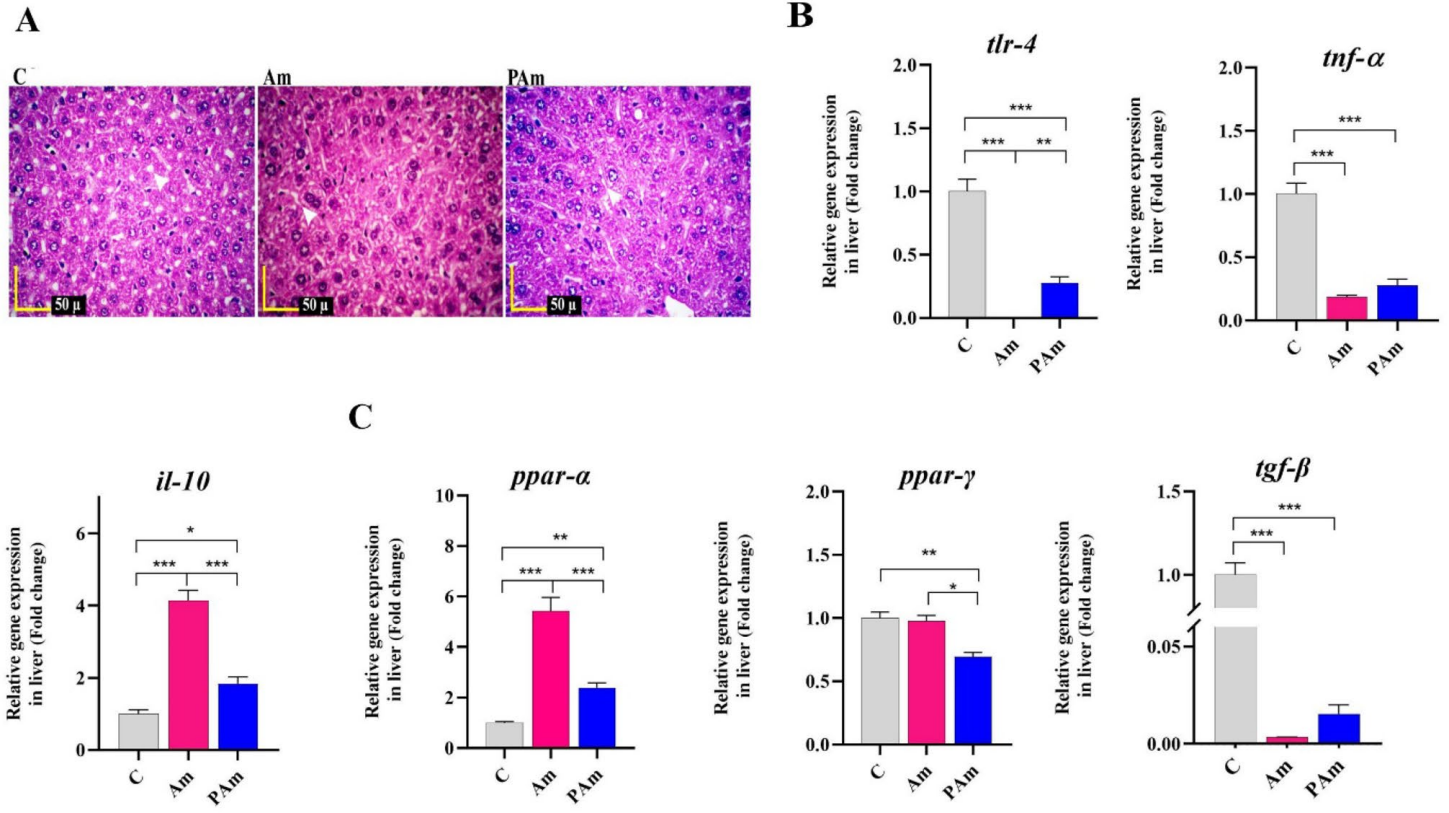
The effect of a live and pasteurized *A. muciniphila* on study's genes in the liver of normal mice. Mice were gavaged with a live and pasteurized *A. muciniphila* (10^9^ CFU) for 5 weeks. (**A**) Histopathology of liver (White Arrows: accumulation of lipids droplets) (4 samples per group). Scale bar is 50 µm. Relative mRNA expression of **(B**) inflammation-related genes (e.g. *tlr-4*, *tnf-α*, and *il-10*) and (**C**) lipid metabolism-related genes (e.g. *ppar-α*, *ppar-,* and *tgf-β*) in the liver of normal diet-fed mice. *, **, *** '*P* < 0.05, *P* < 0. 01, and *P* < 0. 001 were considered statistically significant, respectively. C: normal diet + PBS, Am: normal diet + *A. muciniphila* (10^9^ CFU), PAm: normal diet + pasteurized *A. muciniphila* (10^9^ CFU).

Considering the importance of the liver and its functions in the health, we evaluated the effects of alive and pasteurized *A. muciniphila* to improve liver homeostasis in study groups. In the liver, the hepatocytes and sinusoidal liver cells in Am and PAm groups were similar to control group. Indeed, no accumulation of lipids droplets was observed in both treatments, similar to that control groups (Fig. 3A). The gavage of alive *A. muciniphila* remarkably suppressed the hepatic expression of *tlr-4* gene (*p* value < 0.0001). Moreover, the pasteurized form of the bacterium reduced *tlr-4* expression (*p* value < 0.0001) in mice's liver, notably, the live form had more noticeable effect (*p* value < 0.0001). The expression of *tnf*-α significantly reduced following both treatments, however, there was no significant difference between both of them. Both treatments increased *il-10* expression, while the live form had a greater effect on maintain liver health (*p* value < 0.0001) (Fig. 3B).

Supplementation with *A. muciniphila* significantly regulated the lipid metabolism-related gene in the liver of mice. These results were accompanied by up-regulated *ppar-α* and down-regulated *ppar-γ* and *tgf-β* genes. Alive *A. muciniphila* induced a higher mRNA level of *ppar-α* (*p *value < 0.0001), while pasteurized form had a better effect on lowering *ppar-γ* expression (*p* value < 0.0003). The down-regulation of *tgf-β* was observed after both treatments (Fig. 3C). Taken together, our study elicited that administration of alive and pasteurized *A. muciniphila* by mice resulted in improvement of the lipid metabolism and inflammatory markers in liver tissue.

### Administration of *A. muciniphila* improved adipose health by reducing lipid metabolism and inflammation

**Figure 4:**
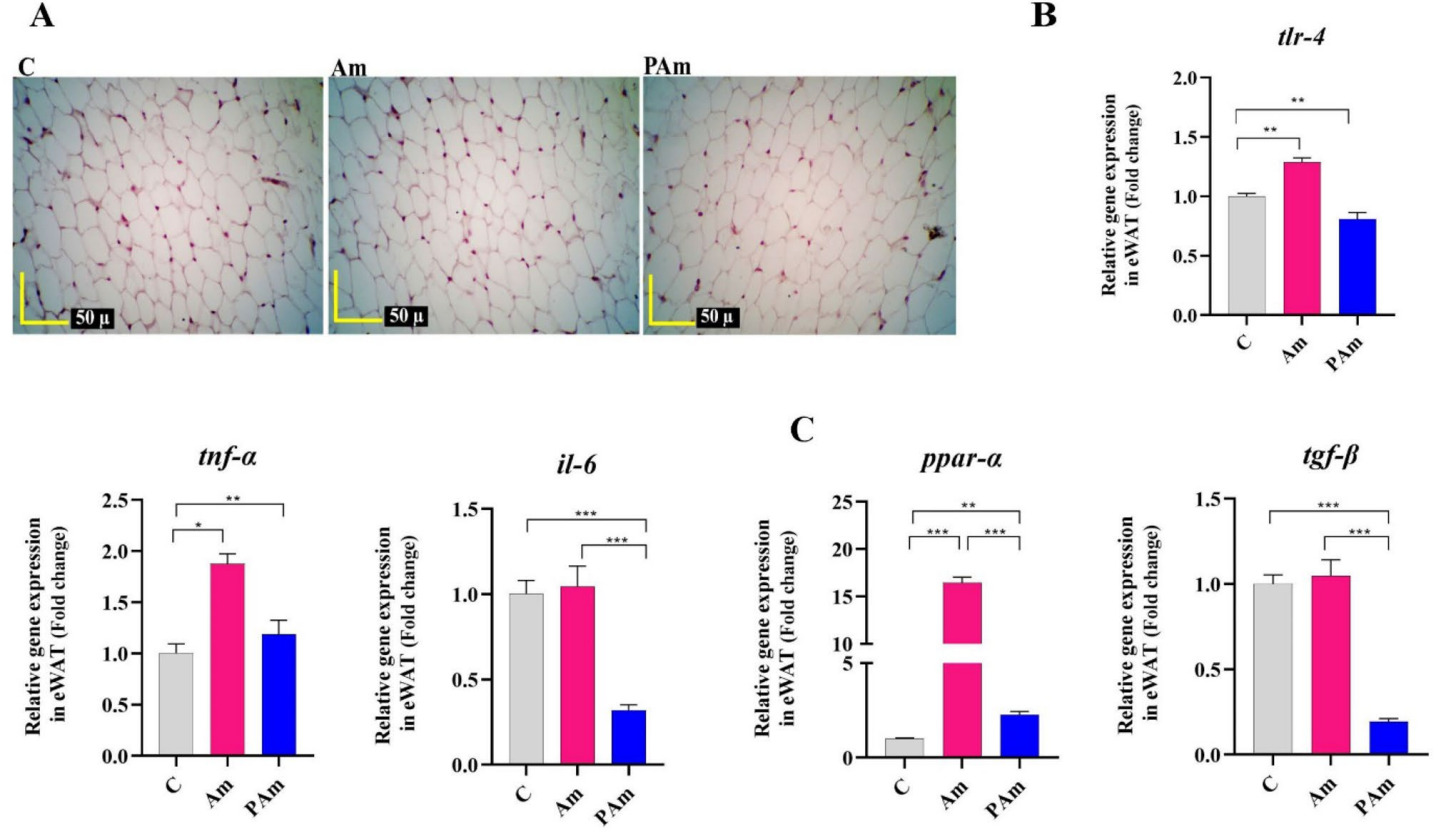
The effect of a live and pasteurized *A. muciniphila* on study's genes in the liver of normal mice. Mice were gavaged with a live and pasteurized *A. muciniphila* (10^9^ CFU) for 5 weeks. (**A)** Histopathology of eWAT of ND-fed mice. (4 samples per group). Scale bar is 50 µm. The mRNA expression of (**B**) inflammation-related genes (e.g. *tlr-4*, *tnf-α*, and *il-6*) and (**C**) lipid metabolism-related genes (e.g. *ppar-α* and *tgf-β*) in the eWAT of ND-fed mice. *, **, *** '*P* < 0.05, *P* < 0. 01, and *P* < 0. 001 were considered statistically significant, respectively. C: normal diet + PBS, Am: normal diet + *A. muciniphila* (10^9^ CFU), PAm: normal diet + pasteurized *A. muciniphila* (10^9^ CFU).

Since inflammation in white adipose tissue is a key factor in the onset of metabolic disorders, therefore*,* alive and pasteurized *A. muciniphila* were administrated to ND-fed mice to determine if they would control and prevent adipo-inflammation to preserve healthy adipose. In histopathology, no change in adipocyte size and no inflammatory infiltration in epididymal white adipose tissue (eWAT) was seen in both treatments, similar to control group (Fig. 4A). A reduction in *tlr-4* and *il-6* mRNA level (*p *value 0.002 and 0.0002, respectively) were seen in PAm group, compared to C group. However, alive *A. muciniphila* had no effect in *il-6* mRNA expression and induced a slight increase in *tlr-4* and *tnf-α* genes expression (Fig. 4B). Interestingly, the mRNA level of *ppar-α* following both treatments were significantly increased, whereas alive *A. muciniphila* induced remarkable upregulation (*p *value < 0.0001). Pasteurized *A. muciniphila* significantly decreased the expression of *tgf-β* (*p *value < 0.0001), while no change was observed in Am group (Fig. 4C). Altogether, probiotic and para-probiotic interventions have led to the maintenance of adipose health by regulating energy balance and immune homeostasis.

### Administration of *A. muciniphila* improved health by modulating some gut microbiota

**Figure 5:**
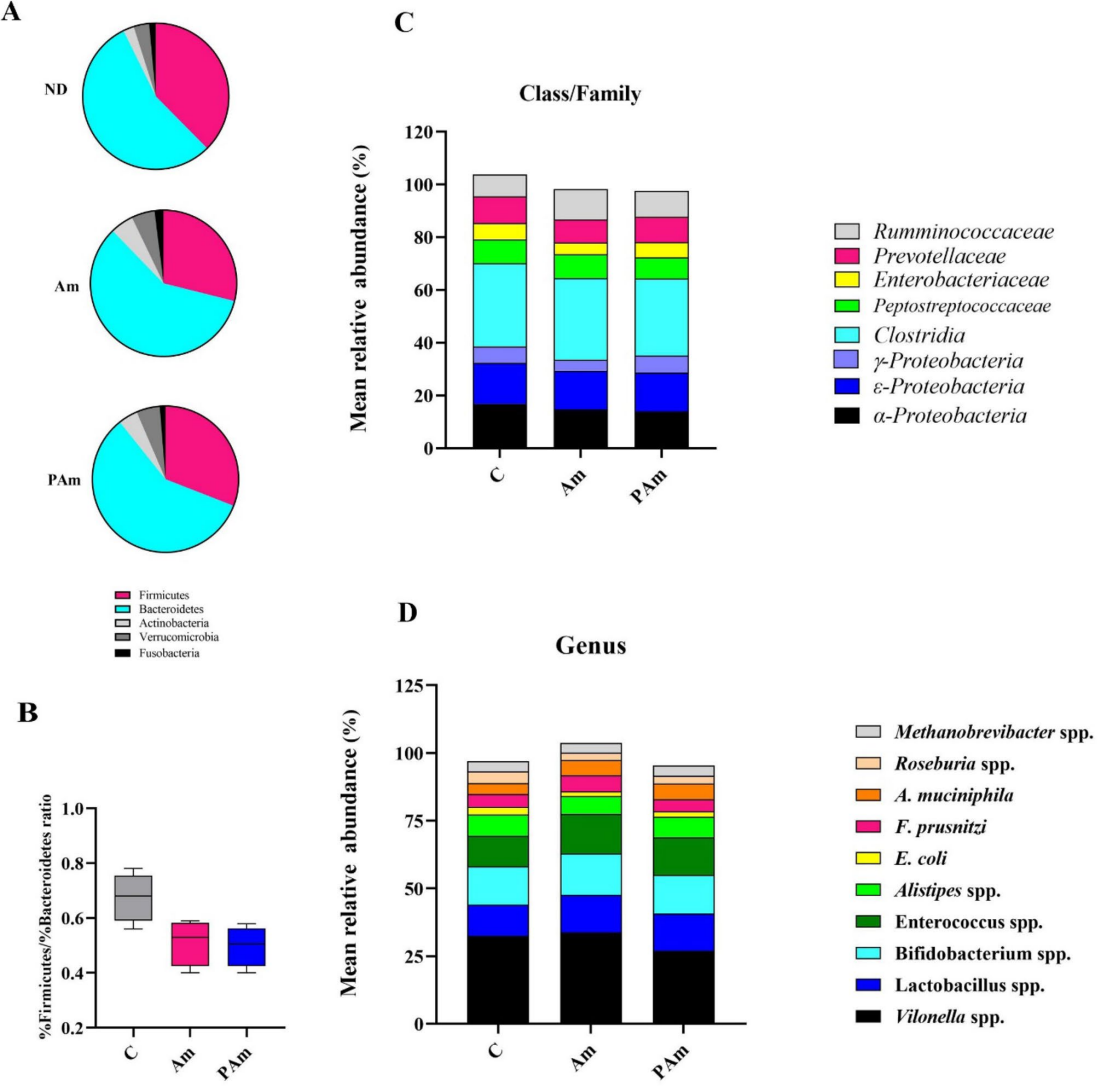
The alter of gut microbiota composition after treated with a live and pasteurized *A. muciniphila* in normal mice. The mean relative abundance at (**A**) phylum. (**B**) Firmicutes to Bacteroidetes ratio. The mean relative abundance at (**C**) Family/Class and (**D**) Genus. N = 10 per group. *p < 0.05. C: normal diet + PBS, Am: normal diet + *A. muciniphila* (10^9^ CFU), PAm: normal diet + pasteurized *A. muciniphila* (10^9^ CFU).

Since normal gut microbiota is essential for maintenance of health and intestinal dysbiosis is capable to inducing several diseases, therefore, the beneficial effects of alive and pasteurized *A. muciniphila* on the gut microbiota pattern was studied. The results showed that alive *A. muciniphila* significantly reduced the abundance of Firmicutes (*p* value 0.02) and increased Bacteroidetes (*p* value 0.01) and Verrucomicrobia (*p* value 0.01), while pasteurized *A. muciniphila* showed no significant change in Phylum level (Fig. 5A). Both treatments displayed some changes in Firmicutes/Bacteroidetes ratio; however, there was no statistical difference was observed (Fig. 5B). A decrease in *Enterobacteriaceae* (*p *value 0.006) and *Prevotellaceae* (*p *value 0.008) abundance in Am group as well as in *α-Proteobacteria* (*p *value 0.02) and *Clostridia* spp. (*p *value 0.005) abundance in PAm group were observed at the Class/Family level relative to control group (Fig. 5C).

At the genus level, the amount of *E. coli* (*p *value 0.02) tended to decrease in Am group, whereas *A. muciniphila* (*p *value 0.01) and *Lactobacillus* spp. (*p* value 0.03) level were significantly increased. Pasteurized *A. muciniphila* induced a significant decrease in *Roseburia* spp. level (*p * value 0.04) (Fig. 5D). However, no significant change in abundance of other genera was observed in both treatment groups. The significant change in the mean relative abundance of gut microbiota between treatment and control groups was shown in Table 2. Overall, results showed that alive *A. muciniphila* treatment can maintain healthy gut microbiota pattern and promote health.

**Table 2:** The The significant change in the mean relative abundance of gut microbiota between treatment and control groups (n = 10 per group).

Microbiota (%)	Study groups			*P* value		
	C	Am	PAm	Am vs. C	PAm vs. C	Am vs. PAm
Firmicutes	37.82 ± 0.590	28.77 ± 0.555	29.43 ± 0.843	**0.02**	0.13	> 0.999
Bacteroidetes	55.79 ± 0.67	58.53 ± 0.57	56.76 ± 0.78	**0.01**	0.83	0.2
Verrucomicrobia	3.58 ± 0.45	5.06 ± 0.37	4.84 ± 0.51	**0.01**	0.14	> 0.999
*α- proteobacteria*	16.9 ± 0.41	14.53 ± 0.49	13.38 ± 0.44	0.53	**0.02**	0.52
*Enterobacteriaceae*	6.42 ± 6.75	4.53 ± 0.44	5.64 ± 0.45	**0.006**	0.38	0.38
*Clostridia*	31.64 ± 0.34	30.60 ± 0.46	29.29 ± 0.46	0.35	**0.005**	0.35
*Prevotellaceae*	10.44 ± 0.60	8.53 ± 0.46	9.49 ± 0.44	**0.008**	0.46	0.34
*E. coli*	2.94 ± 0.42	1.65 ± 0.26	1.81 ± 0.28	**0.02**	0.11	> 0.999
*Lactobacillus* spp.	11.45 ± 0.42	13.59 ± 0.54	13.43 ± 0.40	**0.03**	0.08	> 0.999
*Roseburia* spp.	4.83 ± 0.56	2.65 ± 0.31	2.64 ± 0.30	0.06	**0.04**	> 0.999
*A. muciniphila*	4.09 ± 0.37	5.65 ± 0.39	5.41 ± 0.28	**0.01**	0.14	> 0.999

C: control, Am: *A. muciniphila*, and PAm: pasteurized *A. muciniphila*. Bold P value are indicated statically significant.

## Discussion

The gut microbiota modulates the intestinal immune system, while the immune system affects the composition of the intestinal microbiota. Due to the interaction between the host and the intestinal microbiota, the probiotic bacteria can have significant effects and help the homeostasis of the immune system^28^. Among next-generation probiotics, *A. muciniphila* is widely used for its positive roles in the treatment of various diseases and also for animal and human health preservation^7,17,18,24,25,29^. Moreover in recent years, the propensity towards using non-viable bacterial strains as alternative products to prevent the potential risks of probiotics has increased, especially in high-risk individuals such as infants, the elderly and immunocompromised patients^30^. We carried out a comparative study that compared the effects of alive and pasteurized *A. muciniphila* on normal mice's overall health performance through biochemical, pathological, and molecular techniques. Although several studies addressed the beneficial effects of pasteurized *A. muciniphila* on obesity and metabolic disorders^17, 25, 31^, no research has been performed to identify pharmacological applications of pasteurized *A. muciniphila* for preserving health conditions. This study will help to comprehend the mechanisms and possibility using of pasteurized *A. muciniphila* as an effective para-probiotic in health condition.

Health benefits of probiotics are reported by reducing body and metabolic tissues weight gain as well as preventing fat mass development and liver injury^32,33^. In our study, we demonstrated lowering-body, -liver, and -eWAT weight impact of both forms of *A. muciniphila* in study groups. In consistent with our research, normal mice treated with alive *A. muciniphila* showed a decrease in body and adipose weight gain^7,18^. In addition, several studies showed that alive, pasteurized *A. muciniphila,* its extracellular vesicles, and other derivatives cause a decrease in body weight, metabolism homeostasis, and combating obesity^17,18,34,35^. Besides, normal morphology and structure of vital tissues exist in a healthy body and very important to determine the safety of probiotic administration, while the morphology of the tissues are changed in onset of HFD-induced obesity and metabolic disorders^36,37^, therefore, maintenance of tissue morphology at a normal state seems critical for promoting health. The findings of this research revealed that both forms of *A. muciniphila* could play a positive role in intestinal, liver, and adipose homeostasis, indicating their beneficial effects on health maintenance. Thus, improved metabolic vital tissues structure in *A. muciniphila*-fed mice can be due to positive effects of the probiotic and para-probiotic on digestion, absorption, nutrient usage, and metabolism. In agreement with our pathological results, recent studies confirmed the beneficial health effects of alive *A. muciniphila* on the morphology of liver^7,24^, muscle^7^, colon^12,18^, and adipose tissue of mice undergoing standard diet^18^. In addition, reversing effects of alive or pasteurized *A. muciniphila* on HFD-induced abnormal morphology of tissues revealed in animal models^17,18,24,29^. Taken together, these observations in our and other experiments have shown that the administration of *A. muciniphila* provided some health benefits in multiple tissues, which are correlated with lowering the risk of various diseases.

Increased plasma lipid profiles and inflammatory markers are a major risk factors of cardiovascular disease^38^ and lead to change onset of metabolic disorders^39,40^, therefore, balancing these blood biochemical factors via probiotics or paraprobiotics may help maintain health^41^ and even protect people from diseases^42,43^. In our experiment, we demonstrated that both forms of *A. muciniphila* had corrective properties on lipid, glucose, and liver injury-related enzymes, while the pasteurized form had better effects. These findings are consistent with other recent research demonstrated beneficial lipid-lowering effects of alive *A. muciniphila* in normal mice^7^. Moreover, previous in vivo studies reported the reduction of glucose^18,24^, ALT, and AST after alive *A. muciniphila* administration in ND-fed mice^24^. Furthermore, it is demonstrated that supplementation of *Lactobacillus* and *Bifidobacterium* reduced lipid profiles in healthy adults^41,44^.

One consideration in evaluating the safety of probiotics is the recognition of undesirable changes in immune parameters^45^, owing to emerging evidence that probiotics or their derivatives may have immunomodulatory impacts. The main finding of our cytokine analysis was that *A. muciniphila* induced immunomodulatory effects. Higher level of anti-inflammatory cytokine (IL-10) and lower levels of the pro-inflammatory cytokine (TNF-α and IL-6) were found in the plasma of Am and PAm group, respectively. Regarding, recent investigations exhibited that probiotics improved to maintain balance immunity and inflammation-related cytokines in healthy mice^46^ and subjects^47^. It has been also reported that the live multi-strain probiotics were more effective than the non-viable probiotic strains in in improving insulin resistance associated with intestinal microbiota alteration, butyrate production, and *il*-10 induction^48^. Thus, the possible reasons of better effect of alive *A. muciniphila* on increasing *il*-10 could be due to the production of different components and interactions with other intestinal symbionts. The observations of these investigations support the notion that the consumption of the probiotic and para-probiotic can modify plasma metabolic/inflammation-related profiles intensely and reduce risk of metabolic disorders.

The first tissue exposed to diet-derived nutrients is the gastrointestinal tract, which has cross-talks with other organs by impacting different signaling pathways^49^. Therefore, the improvement of intestinal and immune hemostasis can inhibit inflammatory disease onset, which accompanied by increasing intestinal integrity and preventing passing bacterial components into lamina propria as well as peripheral tissues^50^. Our data demonstrated that alive and pasteurized *A. muciniphila* had a substantial link with intestinal and immune homeostasis, which highlighted their key role in health promotion. We found pasteurized *A. muciniphila* had more beneficial effects on intestinal integrity and no inflammatory adverse effects was seen in non-inflamed Caco-2 and colon of mice. These parameters are critical for determining the intestinal health and immune status of normal mice. In these regards, *A. muciniphila*, which grown in mucin-based medium, induced an increase in intestinal integrity and modulated immune response in ND mice and cell line^18,20,51^. In addition, the beneficial effects of pasteurized *A. muciniphila*^17^ and its extracellular vesicles^18,35^ have been reported on strengthening intestinal integrity in obese mice, which indicates the positive role of non-viable form of this bacterium. Additionally, the upregulation of colonic *il-10*^52^ and increased IL-10 and decreased TNF-α levels in intestinal fluid were observed in probiotics treated-normal animals^53^. Collectively, viable and non-viable forms of probiotics can promote gut health in normal state, while non-viable form was more effective.

Another host factor is involved in the regulation of lipid metabolism and inflammation in the intestine and inversely associated with obesity is Angptl4^54^. Increased Angptl4 can play a key role in maintaining intestinal lipid homeostasis. We found that both treatments had a beneficial effect on lipid metabolism in the colon of mice, similar to Caco-2. It is noteworthy that alive *A. muciniphila* had a greater effect on it, which may be due to generating enzymes or short-chain fatty acids (SCFAs), in addition to surface proteins. Similarly, the greater effect of alive *A. muciniphila* and *Lactobacillus rhamnosus* CNCMI-4317 on *angptl4* expression demonstrated in comparison with *A. municipia's* EVs and heat-killed *L. rhamnosus*, respectively^18,55^. These results suggested that intestinal lipid metabolism is more regulated by viable probiotics. Overall, in our present research and previous studies, these findings support a strong connection between *A. muciniphila* and immune, lipid, and intestinal homeostasis, suggesting direct and indirect effects of the bacterium on the improvement of gut health.

One of the peripheral organs proved that have a strong association with gut is liver, which is in many respects a reflection of a person's health^56^ and has multiple functions and cross-talking with other organs. The improvement of gut health by using probiotics is associated with the restoration and maintenance of liver homeostasis, thereby preventing NAFLD^57^. Recently, probiotics and para-probiotics are widely used for their beneficial role in health and immunity^58,59^. We found that orally administrated both forms of *A. muciniphila* triggered hepatic immune response-/lipid metabolism-related genes in study groups, which indicated improving liver health. Notably, alive *A. muciniphila* had better immunomodulatory and lipid-regulating effects on the liver of mice. In line with these findings, oral daily *A. muciniphila* administration improved liver health by reducing lipid accumulation, downregulating FA oxidation-related genes, and reducing chronic low-grade inflammation in normal mice^7^. Treatment with *Bifidobacterium animalis* induced lipid metabolism increase, while inflammatory genes in healthy rats were unchanged^60^. Moreover, the beneficial hepatic lipid-lowering capability of *A. muciniphila* was demonstrated in ND-fed mice^24^. Altogether, these observations demonstrated the remarkable role of alive and pasteurized *A. muciniphila* in the health of mice's liver, which represents the importance of probiotics and para-probiotics in the maintenance of hepatic lipid and immune homeostasis.

Adipose tissue is an immunological organ that plays a key role in the homeostasis of energy and stores extra lipids^61^. In metabolic diseases, adipose tissue is the first peripheral tissue to be affected by HFD^49^, therefore, restoration and maintenance of healthy adipose can help to maintain metabolic homeostasis. Our results showed oral administration of pasteurized *A. muciniphila* had better effects on the alleviation of adipo-inflammation, while alive form of the probiotic resulted in better effects on lipid homeostasis in the normal adipose tissue. In agreement with our research, the positive role of *A. muciniphila* in the adipose health and immune status in normal^18^ and obese mice^18,62^ were reported. In addition, specific strains of *Lactobacillus* modulate cytokines secretion in mouse adipocyte cell line^63^. These findings may provide new evidence for the administration of probiotics and para-probiotics to metabolic normal mice and also suggested a new strategy to prevent the onset of metabolic diseases such as obesity.

Positive regulation of the gut microbiota has been proposed as one of the mechanisms underlying probiotic effects^64^; nevertheless, contradictory data exists regarding probiotics ability to modulate intestinal microbiota in healthy hosts. For instance, proper effects of probiotics on modulation of beneficial microbiota was shown in previous study^65^, while another study was reported no significant change in gut microbiota compositions after probiotics consuming by healthy individuals^66^. Although previous studies addressed the supplementation of *A. muciniphila* did not induce major changes in gut microbiome in obese human^25^ and HFD-induced obese mice^12^, no data was reported in case of *A. muciniphila's* effects on gut microbiota pattern of ND-fed mice. In our study, we demonstrated that alive *A. muciniphila* was more effective in improving gut microbiota composition, compared to the pasteurized form. In Am group, there was a decrease in amount of Firmicutes, *Enterobacteriaceae*, *Prevotellaceae*, and *E. coli* as well as increased abundance of Bacteroidetes, Verrucomicrobia, *Lactobacillus* spp. and *A. muciniphila*. On the other hand, the significant reduction in *α-Proteobacteria*, *Clostridia*, and *Roseburia* spp. abundance was observed in PAm group. As a result, daily supplementation of *A. muciniphila* generated a reduction in harmful bacteria and an increased population of beneficial microbiota that could be correlated with improving the immune and lipid homeostasis of mice in this study. In these regards, 10-strain probiotic cocktail (i.e. 5 *Lactobacillus* and 5 *Enterococcus* strains) modulate gut microbiota in ND-fed mice by increasing SCFA-producers bacteria^67^. Moreover, we demonstrated that the Firmicutes/ Bacteroidetes (F/B) ratio did not have a significant change following both treatments, while contradictory data have been reported on the F/B ratio, which may be due to many lifestyle-associated factors or methodological differences^68^. Overall, these data demonstrated that consuming probiotics can play a vital role in fostering health via improvement of gut microbiota composition.

In conclusion, the above our outcomes displayed that *A. muciniphila* improved body weight, plasma biochemical and inflammatory markers, and morphology of vital tissues. The administration of both alive and pasteurized *A. muciniphila*, significantly promote gut, adipose, and liver health by modulating immune response and lipid metabolism as well as intestinal homeostasis by improving gut barrier functions and intestine microbiota composition in the study groups. According to better health effects of pasteurized *A. muciniphila*, use of the pasteurized form as a new strategy seems to be a valid, safe, and potentially more cost-effective medication to improve the host's health and reduce the risk of metabolic disorders. This study will help to comprehend the probiotic and para-probiotic mechanism and possibility of using pasteurized *A. muciniphila* as a potent para-probiotic bacterial combination in the health promotion of normal mice.

## Supplementary Information

Supplementary Information.
