# Supplementary material for: Comparative effects of alive and pasteurized Akkermansia muciniphila on normal diet-fed mice
Source: Sci Rep. 2021 Sep 9;11:17898. doi: 10.1038/s41598-021-95738-5 (PMC8429653; doi:10.1038/s41598-021-95738-5)
Supplement: Supplementary file 1 — Supplementary Information. [file 41598_2021_95738_MOESM1_ESM.docx]

**Supplementary Table 1.** Sequence of primers used in qPCR in Caco-2 cell line and mice. m: mice.

| **Primer name** | **Forward primer (5`-3`)** | **Reverse primer (5`-3`)** | **Product size**  **(bp)** |
| --- | --- | --- | --- |
| ***gapdh*** | GGAGCGAGATCCCTCCAAAAT | GGCTGTTGTCATACTTCTCATGG | 197 |
| ***tlr-2*** | TTATCCAGCACACGAATACACAG | AGGCATCTGGTAGAGTCATCAA | 160 |
| ***tlr-4*** | AGACCTGTCCCTGAACCCTAT | CGATGGACTTCTAAACCAGCCA | 147 |
| ***zo-1*** | CAACATACAGTGACGCTTCACA | CACTATTGACGTTTCCCCACTC | 105 |
| ***ocldn*** | AAGAGTTGACAGTCCCATGGCATAC | ATCCACAGGCGAAGTTAATGGAAG | 133 |
| ***cldn-1*** | GCATGAAGTGTATGAAGTGCTTGG | CGATTCTATTGCCATACCATGCTG | 132 |
| ***angptl4*** | ATGCCCAGTACGAACATTTCC | CTGAGTCAAGGGTGCTAAAGC | 135 |
| ***tnf-α*** | ACATCCAACCTTCCCAAACG | GCCCCCAATTCTCTTTTTGAG | 151 |
| ***m-rpl-19*** | TCAGCCACAACATTCTCA | GCACCTCCAACAGTAAGT | 138 |
| ***m-tlr-2*** | TCCTGCGAACTCCTATCC | CCTGGTGACATTCCAAGAC | 151 |
| ***m-tlr-4*** | GCCTTTCAGGGAATTAAGCTCC | GATCAACCGATGGACGTGTAAA | 114 |
| ***m-zo-1*** | GCCGCTAAGAGCACAGCAA | TCCCCACTCTGAAAATGAGGA | 134 |
| ***m-cldn-1*** | TCTGCCACTTCTCACTTCCA | GCCTATACCCTTGCTCTCTGT | 95 |
| ***m-cldn-2*** | CAACTGGTGGGCTACATCCTA | CCCTTGGAAAAGCCAACCG | 128 |
| ***m-ocldn*** | TTGAAAGTCCACCTCCTTACAGA | CCGGATAAAAAGAGTACGCTGG | 129 |
| ***m-il-10*** | GCACTACCAAAGCCACAAG | AGTAAGAGCAGGCAGCATAG | 85 |
| ***m-tnf-α*** | AACAACTACTCAGAAACACAAG | GCAGAACTCAGGAATGGA | 130 |
| ***m-angptl4*** | ACTGTGAGATGACTTCAGATGG | ATTGGCTTCCTCGGTTCC | 174 |
| ***m-hprt*** | TCAGTCAACGGGGGACATAAA | GGGGCTGTACTGCTTAACCAG | 142 |
| ***m-ppar-α*** | CACTTGCTCACTACTGTCCTT | GATGCTGGTATCGGCTCAA | 110 |
| ***m-ppar-γ*** | GGTGCTCCAGAAGATGACAGA | TCAGCGGGTGGGACTTTC | 154 |
| ***m-tgf-β1*** | AATTCCTGGCGTTACCTT | TGTATTCCGTCTCCTTGG | 116 |
| ***m-il-6*** | TCCATCCAGTTGCCTTCT | TAAGCCTCCGACTTGTGAA | 137 |
| ***m-lpl*** | TTCTCCTGATGACGCTGATT | TCACACGGATGGCTTCTC | 218 |

**Supplementary Table 2.** Sequence of primers used for the gut microbiota analysis.

| **Primer name** | **Forward primer (5`-3`)** | **Reverse primer (5`-3`)** | **Product size (bp)** | **Reference** |
| --- | --- | --- | --- | --- |
| **Firmicute** | GGAGYATGTGGTTTAATTCGAAGCA | AGCTGACGACAACCATGCAC | 126 | [1] |
| **Bacteroidetes** | GTTTAATTCGATGATACGCGAG | TTAASCCGACACCTCACGG | 122 | [1] |
| **Actinobacteria** | TGTAGCGGTGGAATGCGC | AATTAAGCCACATGCTCCGCT | 277 | [1] |
| ***Verrucomicrobia*** | TCAKGTCAGTATGGCCCTTAT | CAGTTTTYAGGATTTCCTCCGCC | 97 | [1] |
| **Fusobacteria** | GATCCAGCAATTCTGTGTG | CGAATTTCACCTCTACACTTG | 292 | [2] |
| ***Clostridia*** | AAATGACGGTACCTGACTAA | CTTTGAGTTTCATTCTTGCGAA | 440 | [3] |
| ***γ-Proteobacteria*** | TCGTCAGCTCGTGTYGTGA | CGTAAGGGCCATGATG |  | [4] |
| ***α-Proteobacteria*** | CIAGTGTAGAGGTGAAATT | CCCCGTCAATTCCTTTGAGTT |  | [4] |
| ***ε-Proteobacteria*** | TAGGCTTGACATTGATAGAATC | CTTACGAAGGCAGTCTCCTTA | 189 | [1] |
| ***Enterobacteriaceae*** | CATTGACGTTACCCGCAGAAGAAGC | CTCTACGAGACTCAAGCTTGC | 195 | [5] |
| ***Rumminococcaceae*** | GGCGGC**Y**T**R**CTGGGCTTT | CCAGGTGGAT**W**ACTTATTGTGTTAA | 157 | [6] |
| ***Peptostreptococcus*** | AACTCCGGTGGTATCAGATG | GGGGCTTCTGAGTCAGGTA | 270 | [7] |
| ***Prevotellaceae*** | AACCCGTTGGGTGTGCC | AGIGCCCAAACCTCCATCTCTCC |  | [8] |
| ***Methanobrevibacter* spp.** | CGATGCGGACTTGGTGTTG | TGTCGCCTCTGGTGAGATGTC | 183 | [7] |
| ***Bifidobacterium* spp.** | TCGCGTCYGGTGTGAAAG | CCACATCCAGCRTCCAC | 243 | [9] |
| ***F. prausnitzii*** | GATGGCCTCGCGTCCGATTAG | CCGAAGACCTTCTTCCTCC | 199 | [5] |
| ***A. muciniphila*** | CAGCACGTGAAGGTGGGGAC | CCTTGCGGTTGGCTTCAGAT | 329 | [10] |
| ***Lactobacillus* spp.** | AGCAGTAGGGAATCTTCCA | CACCGCTACACATGGAG | 341 | [9] |
| ***Veillonella* spp.** | ACAACCTGCCCTTCAGA | CGTCCCGATTAACAGAGCTT | 343 | [9] |
| ***Enterococcus* spp.** | CCCTTATTGTTAGTTGCCATCATT | ACTCGTTGTACTTCCCATTGT | 144 | [9] |
| ***Alistipes* spp*.*** | TTAGAGATGGGCATGCGTTGT | TGAATCCTCCGTATTACCGCG | 320 | [3] |
| ***E. coli*** | CATTGACGTTACCCGCAGAAGAAGC | CTCTACGAGACTCAAGCTTGC | 190 | [5] |
| ***Roseburia* spp.** | TACTGCATTGGAAACTGTCG | CGGCACCGAAGAGCAAT | 230 | [11] |
| **Universal** | AAACTCAAAKGAATTGACGG | CTCACRRCACGAGCTGAC | 136 | [1] |

**Reference**:

1. Yang, Y.-W., et al., *Use of 16S rRNA gene-targeted group-specific primers for real-time PCR analysis of predominant bacteria in mouse feces.* Applied and environmental microbiology, 2015. **81**(19): p. 6749-6756.

2. Hermann-Bank, M.L., et al., *The Gut Microbiotassay: a high-throughput qPCR approach combinable with next generation sequencing to study gut microbial diversity.* BMC genomics, 2013. **14**(1): p. 1-14.

3. Vigsnæs, L.K., et al., *Gram-negative bacteria account for main differences between faecal microbiota from patients with ulcerative colitis and healthy controls.* Beneficial microbes, 2012. **3**(4): p. 287-297.

4. De Gregoris, T.B., et al., *Improvement of phylum-and class-specific primers for real-time PCR quantification of bacterial taxa.* Journal of microbiological methods, 2011. **86**(3): p. 351-356.

5. Bartosch, S., et al., *Characterization of bacterial communities in feces from healthy elderly volunteers and hospitalized elderly patients by using real-time PCR and effects of antibiotic treatment on the fecal microbiota.* Applied and environmental microbiology, 2004. **70**(6): p. 3575-3581.

6. Omar, J.M., et al., *Lactobacillus fermentum and Lactobacillus amylovorus as probiotics alter body adiposity and gut microflora in healthy persons.* Journal of functional foods, 2013. **5**(1): p. 116-123.

7. Verma, R., et al., *Real-time analysis of mucosal flora in patients with inflammatory bowel disease in India.* Journal of clinical microbiology, 2010. **48**(11): p. 4279-4282.

8. Jiang, X., et al., *The Microbiome–Metabolome Response in the Colon of Piglets Under the Status of Weaning Stress.* Frontiers in Microbiology, 2020. **11**: p. 2055.

9. Rinttilä, T., et al., *Development of an extensive set of 16S rDNA‐targeted primers for quantification of pathogenic and indigenous bacteria in faecal samples by real‐time PCR.* Journal of applied microbiology, 2004. **97**(6): p. 1166-1177.

10. Schneeberger, M., et al., *Akkermansia muciniphila inversely correlates with the onset of inflammation, altered adipose tissue metabolism and metabolic disorders during obesity in mice.* Scientific reports, 2015. **5**: p. 16643.

11. Larsen, N., et al., *Gut microbiota in human adults with type 2 diabetes differs from non-diabetic adults.* PloS one, 2010. **5**(2): p. e9085.
